# Supplementary material for: Investigation on the Curing and Thermal Properties of Epoxy/Amine/Phthalonitrile Blend
Source: Materials (Basel). 2024 Sep 7;17(17):4411. doi: 10.3390/ma17174411 (PMC11395949; doi:10.3390/ma17174411)
Supplement: Supplementary file 1 [file materials-17-04411-s001.zip › materials-3183865-supplementary.pdf]

materials

Supplementary document

Investigation on the curing and thermal properties of epoxy/amine/phthalonitrile blend

The TGA results of the cured BAPH and BAPH containing 5% DDS were shown in Fig. S1.The curing procedure of the pure BACH was 230 °C/2h, 280 °C/3h, 320 °C/4h, 370 °C/4h according to relevant research [1].The curing procedure of the BAPH containing 5% DDS was 220°C/2h, 270°C/6h based on the results shown in Fig. S2. The pyrolysis features of these two material are almost the same indicating the similar crosslinking structure of the two materials. Moreover, the DDS monomer will decompose if it is dissociative in the resin. Considering the very similar TGA curves it can be inferred that there is chemical bonding between DDS and the poly-phthalonitrile structure.

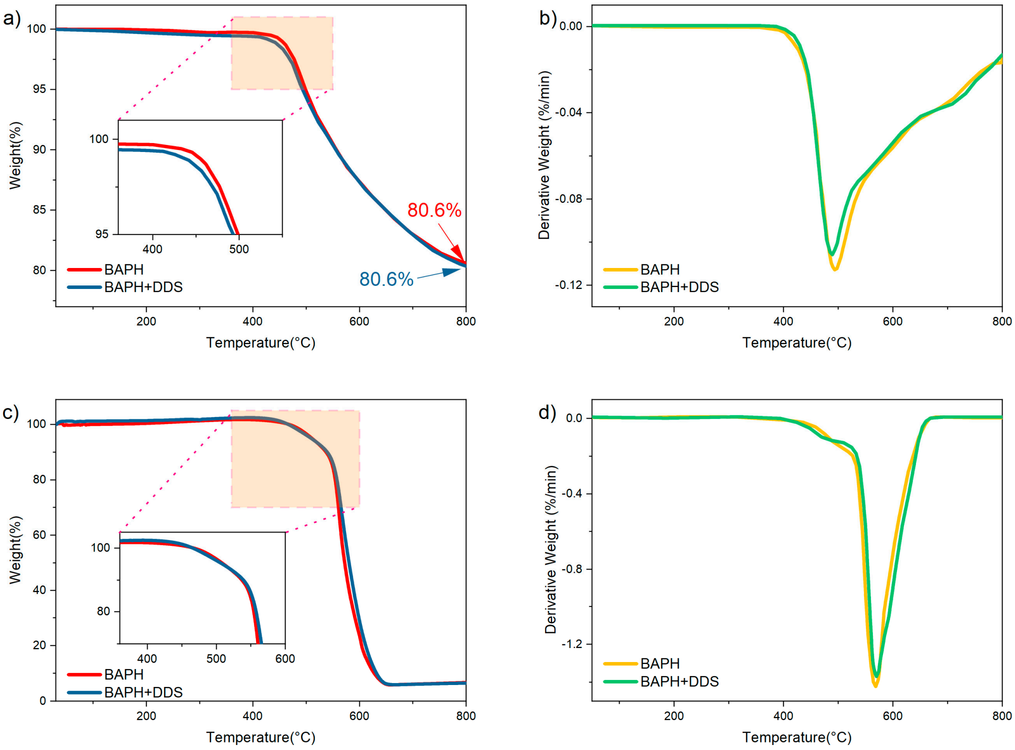

Figure S1. TG (a, c) and DTG (b, d) curves of the BAPH and BAPH/DDS thermosets.

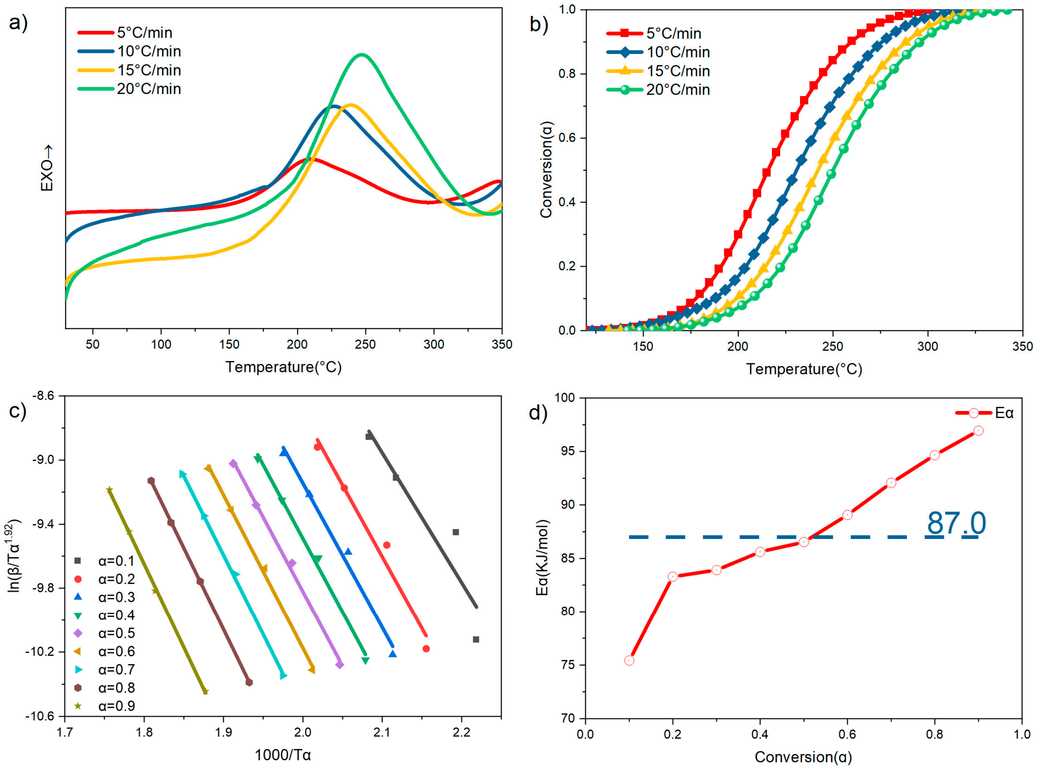

Figure S2. DSC curves of E51/DDS mixture with different heating rates (a), conversion rate *α* as function of reaction (b), linear fitting plots at various conversion rates (c) and the values of E_α depending on *α* for crosslinking reaction (d).

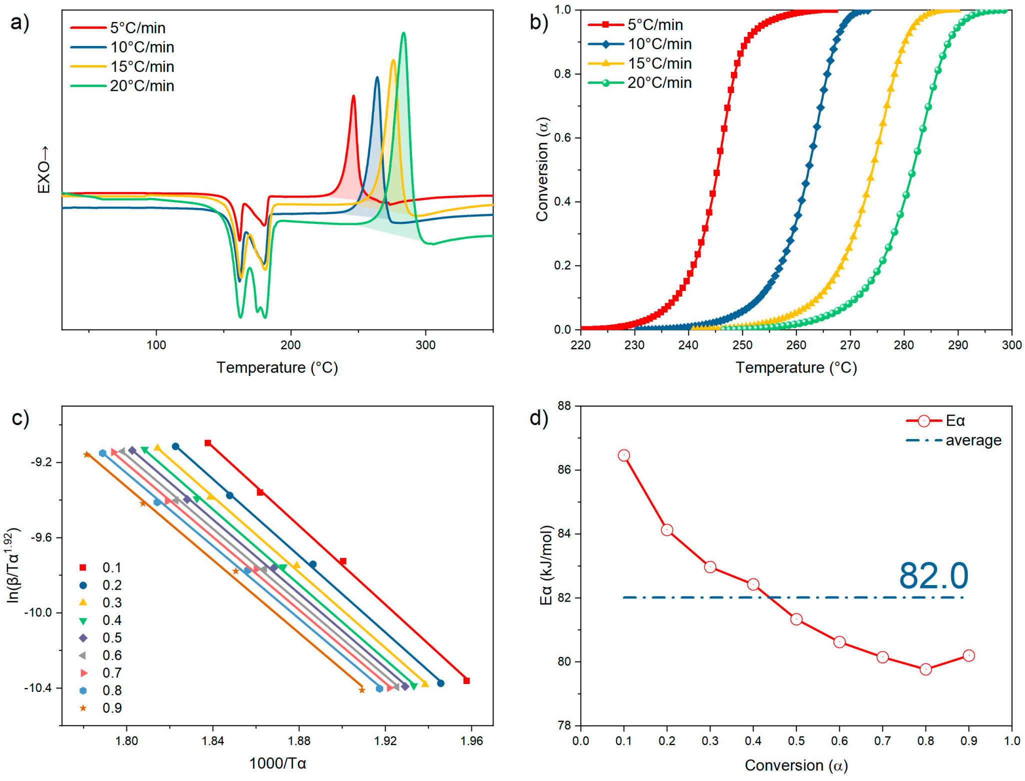

Figure S3. DSC curves of BAPH/5 wt. %DDS with different heating rates (a), conversion rate *α* as function of reaction (b), linear fitting plots at various conversion rates (c) and the values of E_α depending on *α* for crosslinking reaction (d).

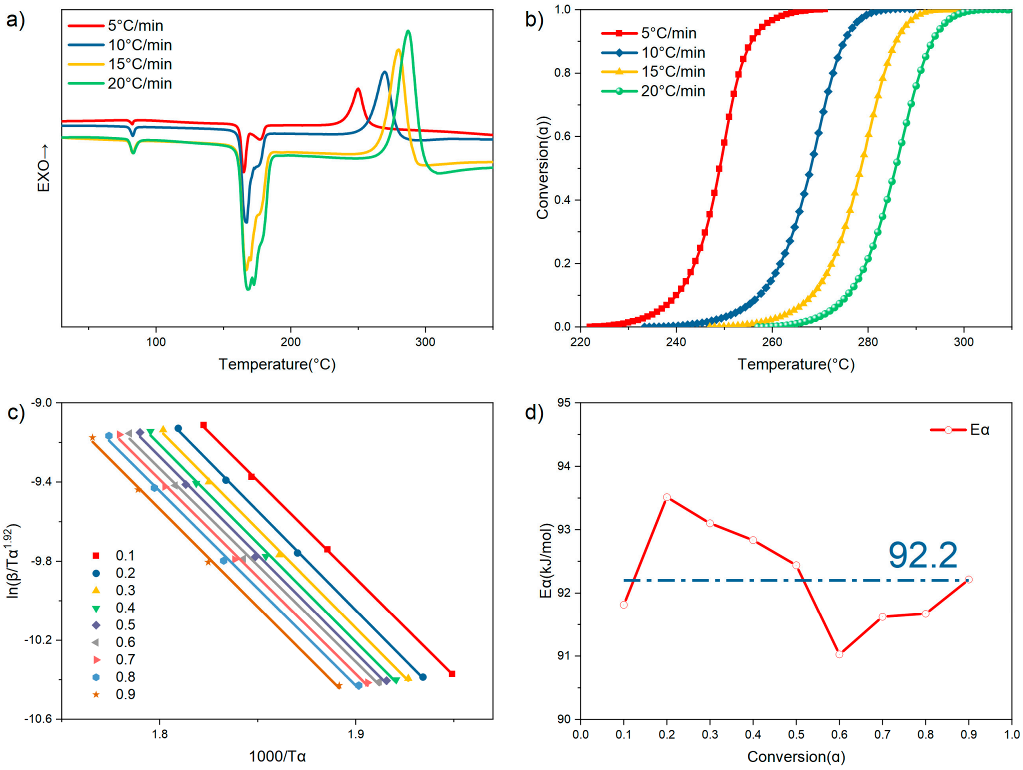

Figure S4. DSC curves of BAPH/10 wt. %DDS with different heating rates (a), conversion rate α as function of reaction (b), linear fitting plots at various conversion rates (c) and the values of E_α depending on α for crosslinking reaction (d).

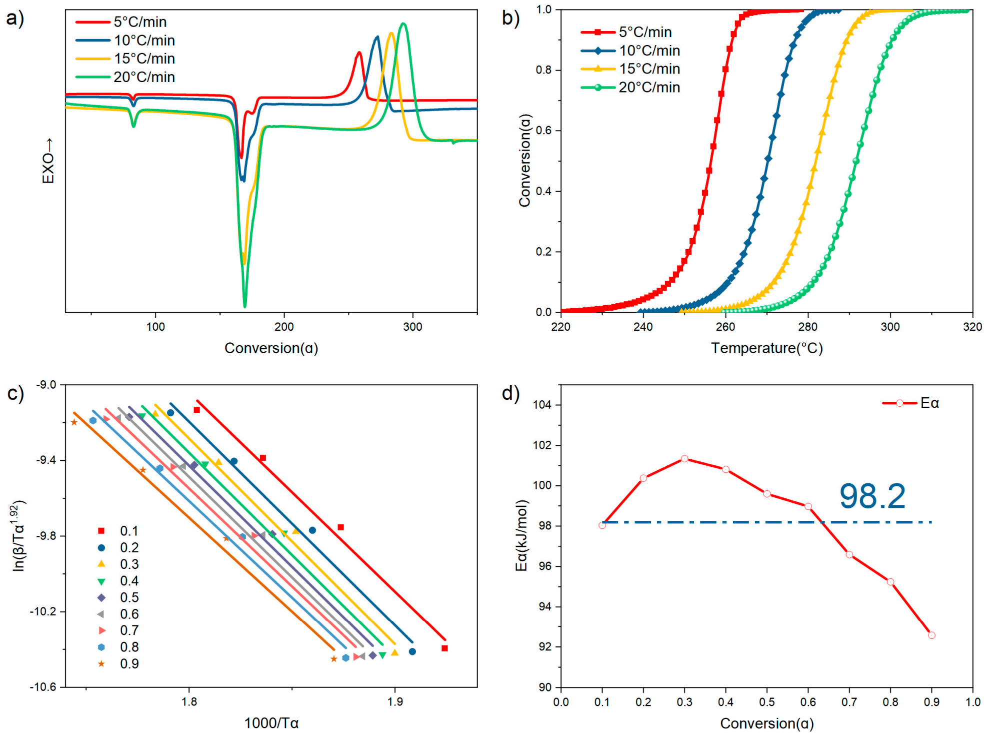

Figure S5. DSC curves of BAPH/15 wt. %DDS with different heating rates (a), conversion rate α as function of reaction (b), linear fitting plots at various conversion rates (c) and the values of E_α depending on α for crosslinking reaction (d).

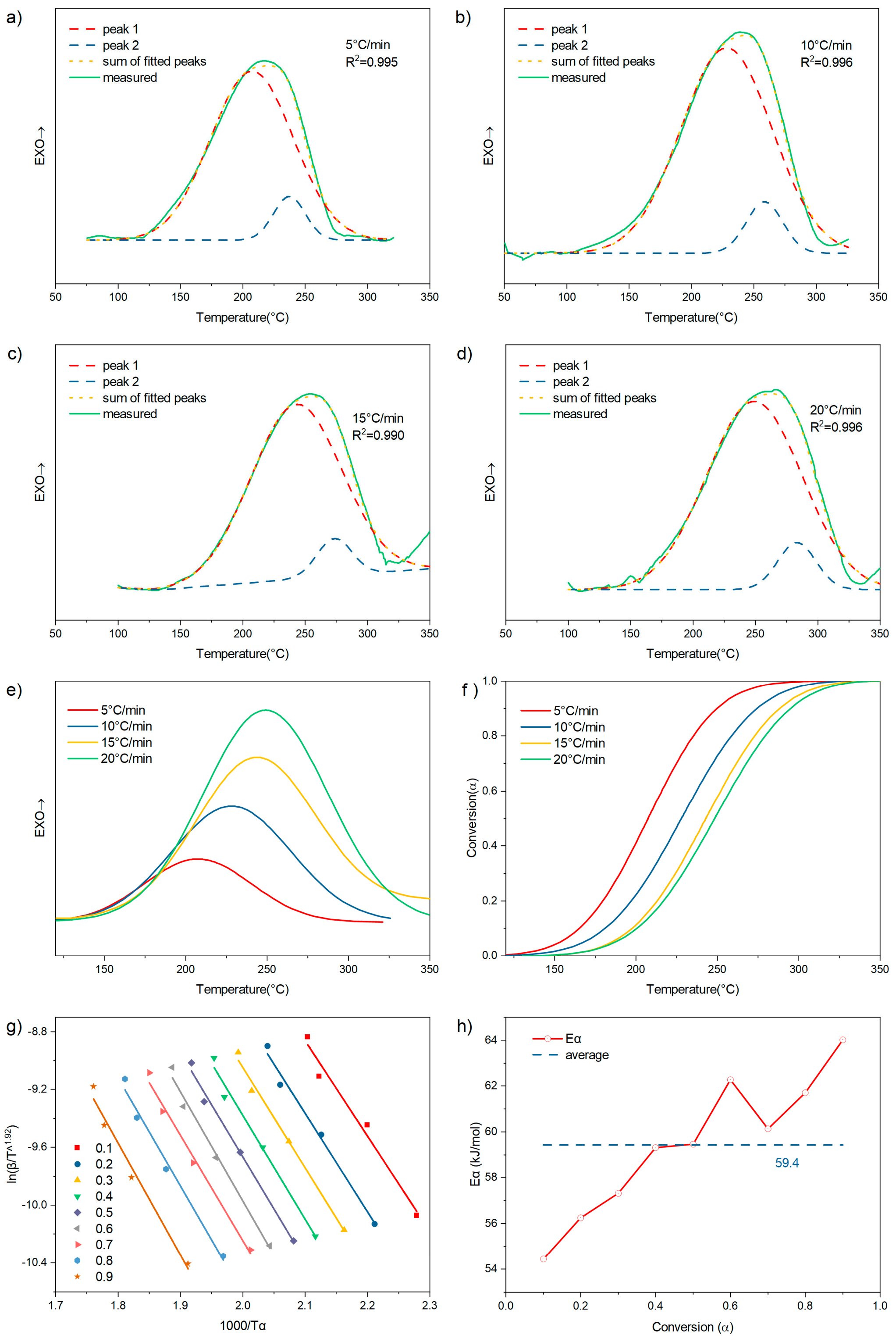

Figure S6. DSC curves of EDPH1 with different heating rates (a-d), the fitted DSC curves of epoxy/DDS curing reaction (e), conversion rate α as function of temperature (f), linear fitting plots at various conversion rates (g) and the values of Eα depending on α for the crosslink reaction (h).

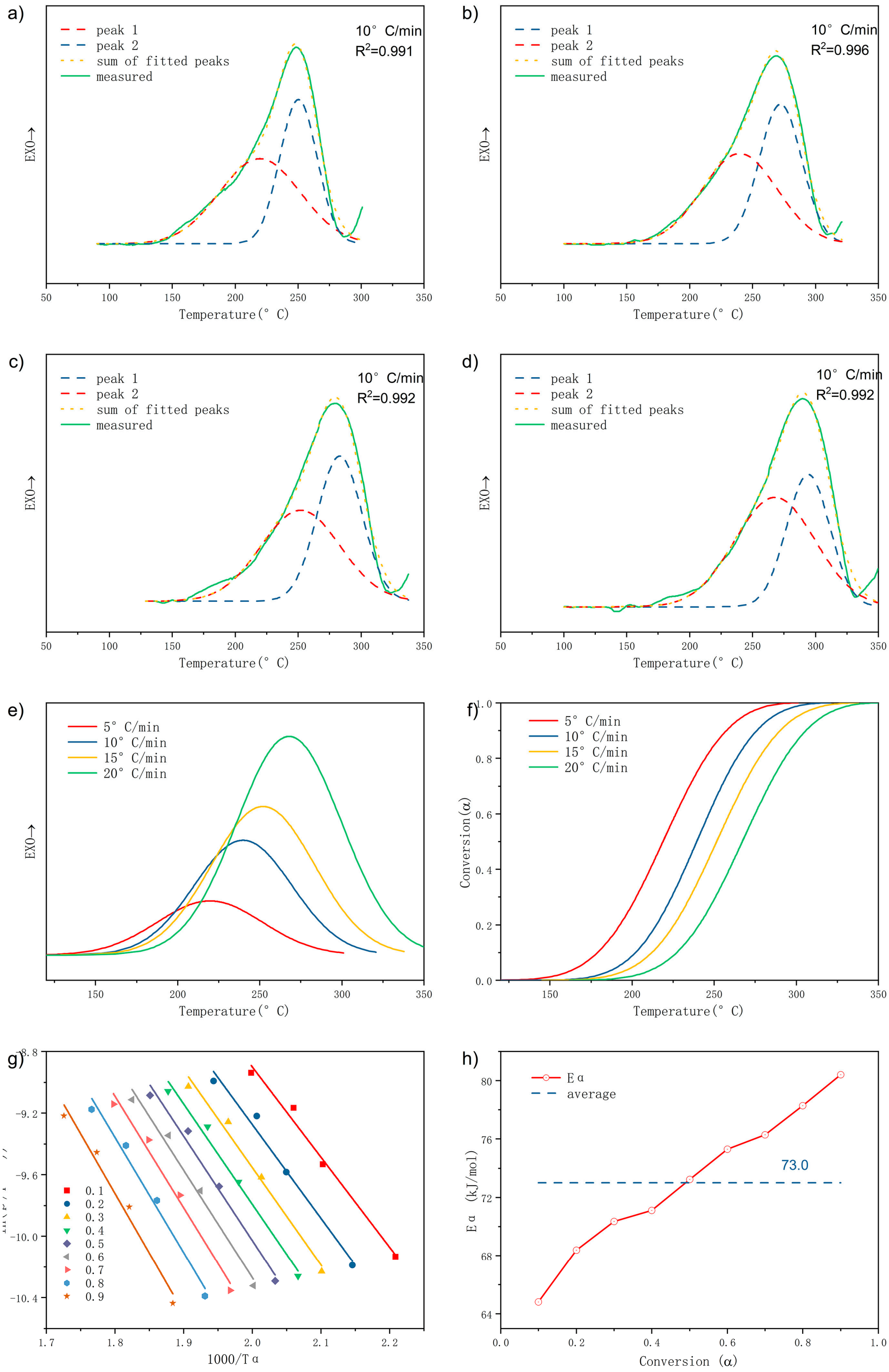

Figure S7. DSC curves of EDPH3 with different heating rates (a-d), the fitted DSC curves of epoxy/DDS curing reaction (e), conversion rate α as function of temperature (f), linear fitting plots at various conversion rates (g) and the values of Eα depending on α for the crosslink reaction (h).

[1] T.M. Keller, D.D. Dominguez, High temperature resorcinol-based phthalonitrile polymer, Polymer 46 (2005) 4614–4618. https://doi.org/10.1016/j.polymer.2005.03.068.
